# The complete chloroplast genome of *Angelica sylvestris*, the type species of the genus *Angelica* (Apiaceae)

**DOI:** 10.1080/23802359.2019.1677522

**Published:** 2019-10-18

**Authors:** Chenyang Liao, Xiaofu Chen, Yi Chen, Yundong Gao

**Affiliations:** a College of Architecture and Environment, Sichuan University, Chengdu, Sichuan, P. R. China;; b Department of Plant Biology, University of Illinois at Urbana and Champaign, Urbana, IL, USA;; c Key Laboratory of Mountain Ecological Restoration and Bioresource Utilization, Chengdu Institute of Biology, Chinese Academy of Sciences, Chengdu, Sichuan, P. R. China

**Keywords:** *Angelica*, Apioideae, chloroplast, genome

## Abstract

*Angelica sylvestris* L. is the type species of the genus *Angelica* L., which was considered as one of the largest and most complicated groups in the family Apiaceae. The complete chloroplast genome of *A. sylvestris* was determined for the first time, and revealed a circle quadripartite structure of 146,910 bp in length comprising a large single-copy region (LSC) of 93,503 bp, a small single-copy region (SSC) of 17,833 bp and a pair of inverted regions (IRs) of 17,787 bp each. Based on the reported chloroplast genomes of Apioideae, phylogenetic analyses suggested that *A. sylvestris* was located in the *Angelica* group together with most *Angelica* species, which coincided with previous molecular systematic research.

*Angelica* L. is a large genus of the family Apiaceae that approximately comprises more than 100 species around the world (Shan 1992; Sheh et al. 2005). It was established by Carl von Linné in 1753 and typed with *A. sylvestris* L. (Linnaeus 1753). As the most widely distributed species, *A. sylvestris* mainly occurs in Europe and North Atlantic Islands, and also appears in Central Asia. *Angelica* species exhibit high diversities and variations in morphology, resulting in extreme difficulties in distinguishing and identifying the species. Recent studies revealed the benefits of DNA sequence data for phylogenetic and taxonomic analysis of *Angelica* species and its allies (Xue et al. 2007; Liao et al. 2013). Herein, we examined the chloroplast genome of *A. sylvestris* using next-generation sequencing (NGS), which will be essential to the research on the genus *Angelica* and allied taxa, and provide significant information for identification, phylogeny, and evolution.

Fresh leaves of *A. sylvestris* were collected from the greenhouse of University of Illinois at Urbana and Champaign, Urbana, Illinois, USA (40°5′21″E; 88°12′46″N), the voucher specimens (accession no. IL4581) and DNA samples were deposited in the University of Illinois Herbarium. Morphological characteristics are measured using Karyotype (Altınordu et al. 2016). Total genomic DNA was extracted by Plant Genomic DNA Kit. The isolated genomic DNA was manufactured to average 400 bp paired-end (PE) library using the Illumina Hiseq platform (Illumina, San Diego CA), and then sequenced by Illumina genome analyzer (Hiseq PE150). The chloroplast genome of *A. sylvestris* was mapped and reconstructed using Geneious Prime 2019.1.1 (Kearse et al. 2012) and annotated with *Angelica polymorpha* (GenBank: NC041580.1) as the reference. The complete chloroplast genome was deposited in GenBank with accession no. MN275034.

The complete chloroplast genome of *A. sylvestris* was 146,910 bp long in a circular form, which consisted of four distinct regions: the large single-copy region (LSC) of 93,503 bp, the small single-copy region (SSC) of 17,833 bp and two copies of inverted regions (IRs) of 177,787 bp each. The total GC content of *A. sylvestris* was 38%, with 36% for the LSC, 31% for the SSC and 45% for each IR. The genome contained 4 rRNAs, 29 tRNAs, and 84 protein-coding genes, for a total of 125 genes. The tRNA coding genes were mainly distributed in the LSC (including 22), six in each IR and one in the SSC, while the rRNA coding genes were only located in the IRs.

The phylogenetic analysis was conducted between *A. sylvestris* and the related species in Apioideae. The complete chloroplast genome sequences of 20 species from 11 genera were aligned using MAFFT (Katoh et al. 2002) and trimmed properly using trimAl v1.4 (Capella-Gutierrez et al. 2009). A neighbor-joining (NJ) tree was performed using MEGA7.0 (Kumar et al. 2016) with 1000 bootstrap replicates, which are shown next to the branches in Figure 1. The result suggested that *A. sylvestris* was located in the *Angelica* group and associated with most *Angelica* species and *Pimpinella rhomboidea* var. *tenuiloba*, which coincided with previous studies on morphological and molecular evidence (Liao et al. 2012, 2013).

**Figure 1.**
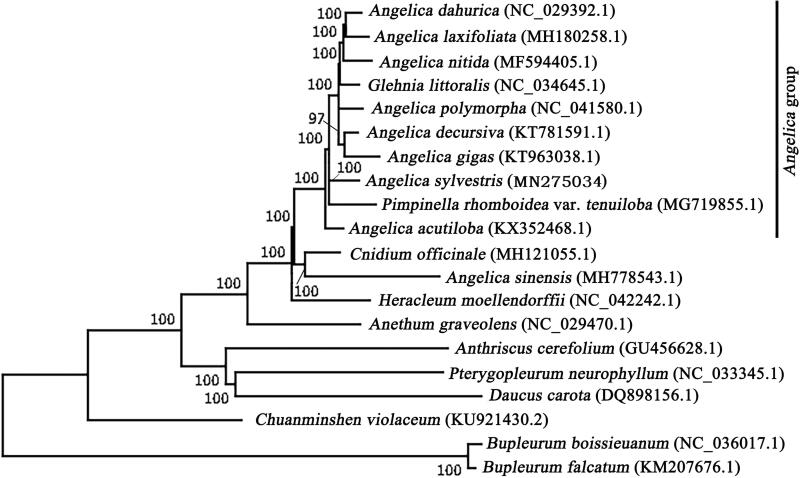
Neighbour-joining tree of *Angelica sylvestris* and related species based on complete chloroplast genome sequences. Numbers on the nodes show the bootstrap values from 1000 replicates.
